# Hyaluronic Acid‐Guided Cerasome Nano‐Agents for Simultaneous Imaging and Treatment of Advanced Atherosclerosis

**DOI:** 10.1002/advs.202202416

**Published:** 2022-12-18

**Authors:** Qian Ma, Sijing Wu, Ling Yang, Yaohua Wei, Chaoyong He, Wenshan Wang, Yingxin Zhao, Zhijian Wang, Shiwei Yang, Dongmei Shi, Yuyang Liu, Zhiming Zhou, Jiefang Sun, Yujie Zhou

**Affiliations:** ^1^ Department of Cardiology Beijing Anzhen Hospital Capital Medical University 100029 Beijing P. R. China; ^2^ Beijing Key Laboratory of Precision Medicine of Coronary Atherosclerotic Disease Clinical Center for Coronary Heart Disease Capital Medical University Beijing 100029 P. R. China; ^3^ Beijing Inno Medicine Co. Ltd. Beijing 100195 P. R. China; ^4^ Beijing Anzhen Hospital Beijing Institute of Heart Lung and Blood Vessel Disease Beijing 100029 P. R. China

**Keywords:** active targeting delivery, atherosclerosis, cerasomes, diagnosis and treatment, hyaluronic acid

## Abstract

Early noninvasive screening and regression therapy for vulnerable atherosclerotic plaques remain challenging. In this study, it is aimed to develop a new approach for the active targeting of atherosclerotic plaques with nano‐agents to aid imaging and treatment. Biocompatible hyaluronic acid (HA)‐guided cerasomes are generated to selectively target CD44‐positive cells within the plaque in in vitro studies and in vivo testing in Apoe^−/−^ mice. Rosuvastatin (RST) is encapsulated in the HA‐guided cerasome nano‐formulation to produce HA‐CC‐RST, which results in significant plaque regression as compared to treatment with the free drug. Moreover, gadodiamide‐loaded HA‐CC enhances magnetic resonance images of vulnerable plaques, thereby attaining the goal of improved simultaneous treatment and imaging. Transcriptomic analysis confirms plaque regression with HA‐CC–RST treatment, which potentially benefits from the anti‐inflammatory effect of RST. In summary, a safe and efficient nano‐formulation for the targeted delivery of active agents to atherosclerotic plaques is developed and may be applicable to other diagnostic and therapeutic agents for atherosclerosis treatment.

## Graphical abstract

A safe and efficient hyaluronic acid guided cerasomes is generated for the targeted delivery of active agents to atherosclerotic plaques, which might be attractive diagnostic and therapeutic agents for atherosclerosis treatment.

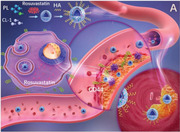

## 1 Introduction

Atherosclerosis is the major pathological contributor to cardiovascular disease (CVD), which causes more than 17.5 million global deaths each year and threatens quality of life and financial security.^[^ ^1^, ^2^ ^]^ Clinical studies have shown that 70–80% of major adverse cardiovascular events (MACEs) is caused by rupture of unstable atherosclerotic plaques.^[^ ^3^ ^]^ Inflammation, accumulation of cholesterol, and extracellular matrix debris in the arteries all contribute to this condition. Current therapies include low‐density lipoprotein (LDL) cholesterol‐lowering drugs, such as statins and PCSK9 inhibitor (PCSK9i),^[^ ^4^ ^]^ which delay atherosclerosis without precipitating plaque regression^[^ ^5^ ^]^ and are often accompanied by adverse reactions, such as hepatoxicity and myopathy.^[^ ^6^ ^]^ For example, PCSK9i substantially decreased plasma cholesterol level but caused ≈2% plaque regression.^[^ ^4^ ^]^ Clinical diagnosis relies on intravascular ultrasound or laser coherence tomography, which are invasive, expensive, and labor‐intensive, precluding the large‐scale screening of high‐risk patient populations.^[^ ^7^ ^]^ These shortcomings illustrate the need to develop sensitive imaging probes for the noninvasive detection of vulnerable plaques.

The utility of actively‐targeted nano‐formulations delivered by direct infiltration through the injured endothelium or dysfunctional adventitial neovessels to elicit local accumulation of drugs has been demonstrated, along with enhanced pharmacokinetics for treatment and plaque imaging.^[^ ^5^, ^6^, ^7^, ^8^, ^9^, ^10^ ^]^ However, uncertainty over safety and difficulties in industrial production have hindered progress. Recently, hybrid liposomal cerasomes (CCs) have been developed as novel drug‐delivery systems.^[^ ^11^, ^12^, ^13^ ^]^ Owing to their silica‐like surfaces, CCs have the advantage of outstanding morphological stability compared to that of liposomes and excellent biocompatibility. The drug loading method of CCs is similar to that of liposomes, which means that various active agents can be flexibly loaded into its aqueous core or lipid bilayer. Cluster determinant 44 (CD44) is a cell‐surface hyaluronan receptor with physiological functions in hematopoiesis, angiogenesis, cell differentiation, proliferation, and migration. It has been shown to be upregulated and activated in vascular endothelial cells, smooth muscle cells, and macrophages within atherosclerotic plaques compared to that in normal vessels.^[^ ^14^, ^15^ ^]^ Hyaluronic acid (HA), a natural ligand of CD44, shows excellent biocompatibility, biodegradability, and low immunogenicity. Therefore, actively targeting nano‐formulations bearing CD44‐specific HA ligands should selectively accumulate in atherosclerotic plaques.^[^ ^16^, ^17^ ^]^

**Scheme 1:**
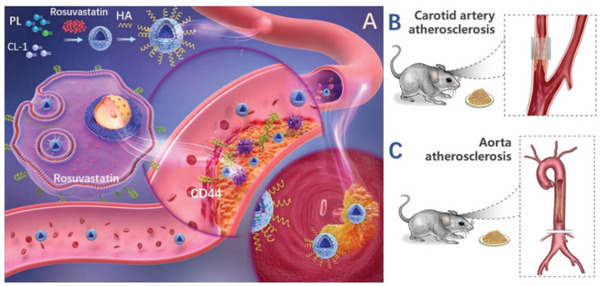
A) Illustration of HA‐CC targeting the atherosclerotic plaque in vivo. B) Left carotid artery atherosclerosis, and C) aortic atherosclerosis model of Apoe^−/−^ mice.

Here, we aimed to develop an HA‐modified CC (HA‐CC) for the targeted delivery of anti‐atherosclerotic drugs with potential for diagnosis and treatment (**Scheme** **1**). The HA‐CCs were generated to selectively target CD44‐positive cells within the plaque in in vitro and in vivo studies. The HA‐CC nanocarrier showed superior targeting of vulnerable plaques than unmodified CC and could be loaded with gadodiamide (Gd) for noninvasive magnetic resonance imaging (MRI) screening. Rosuvastatin (RST) loaded HA‐CC showed significantly higher local enrichment in CD44‐over‐expressing plaques than free RST, leading to substantial plaque regression. Pharmacological analysis revealed that levels of pro‐inflammatory factors and foam cells decreased, and the transcriptomic analysis after HA‐CC–RST treatment in model mice showed upregulation of anti‐inflammatory genes (CCL5, CCR6, and CXCR5). Thus, RST may exhibit pleiotropy in vivo after targeted delivery. The specific targeting and biological safety of HA‐CC make it a powerful tool for diagnosing and treating high‐risk patients with atherosclerosis.

## 2 Results and Discussion

### 2.1 Selecting HA‐CC Nano‐Vesicles for Loading Active Substances

**Figure 1:**
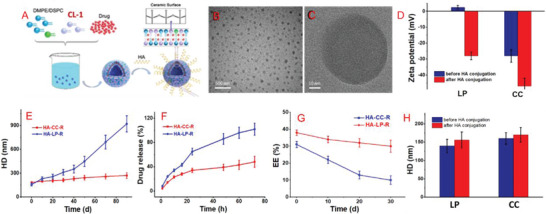
Characterizations and properties of hyaluronic acid‐modified cerasome (HA‐CC) nano‐formulation. A) The schematic diagram for preparation of drug‐loaded HA‐CC–rosuvastatin (RST); B) TEM image of HA‐CC–RST and C) its zoom‐in image; D) Zeta potential of the CC–RST before and after HA conjugation; E–G) Comparison of in vitro properties of the HA‐CC–RST and HA‐LP–RST; H) Dynamic light scattering results of the CC–RST before and after HA conjugation. Data represent the mean ± SD (*n* = 3). Significant differences were not calculated in the qualitative analysis.

Nano‐formulations should exhibit stability along with targeted drug release and biosafety. The CCs were designed to have superior encapsulation capacity, especially for drugs slightly soluble in water. In this study, RST was loaded into the CC membrane layer using a thin‐film hydration method, and hydrophilic Gd was encapsulated in the inner water phase to constitute the CCs. Both components were loaded by passive encapsulation and drug‐loaded HA‐CC was derived after sol–gel cross‐linking (**Figure** **1**A). The TEM images showed good dispersion and uniform spherical morphologies of HA‐CC–RST (Figure 1B,C), with slightly higher contrast of the outer layers than that of the inner layers, indicating a hollow structure. Taking CC–RST as a typical example, before and after HA conjugation, CC–RST was observed to be well dispersed in ultrapure water and had an average hydrodynamic diameters (HD) of ≈160 and ≈170 nm, respectively, determined using dynamic light scattering analysis (Figure 1H). The EE was ≈50%, with a mass ratio of drug to lipid being 1:10, an improvement as compared to other passive loading liposome formulations (Figure 1G). Moreover, the silicon hydroxyl‐rich surface of HA‐CC–RST showed a higher negative charge than CC–RST, with a *ζ* potential of nearly −46.3 eV at pH 7.2, indicating successful modification and good colloid stability (Figure 1D). The CC–RST and HA‐CC–RST had nanostructures and characteristics similar to those of the corresponding modified liposomal analogs, that is, LP–RST and HA‐LP–RST (Figure 1D,H) but had remarkably enhanced long‐term stability and encapsulation capacity. The size of HA‐CC–RST only increased slightly from 178 nm to 260 nm without apparent phase separation during 90 days of storage in water, whereas HA‐LP–RST showed distinct aggregation (Figure 1E). The EE% decreased slowly from 56% in the freshly prepared sample to 43% after 30 days of storage for HA‐CC–RST but showed a rapid decrease from 32% to 10% for HA‐LP–RST (Figure 1F), demonstrating its good stability compared to that of its liposomal counterpart (HA‐LP–RST). Furthermore, HA‐CC–RST demonstrated initial fast drug release, attaining 15% in the first 2 h, followed by a slower release rate such that 40% RST remained inside the HA‐CC after 72 h (Figure 1F). In contrast, drug release by HA‐LP–RST was rapid, and almost 100% of encapsulated RST was released within 72 h. All these results indicated that the inorganic polysiloxane network on the surface of the CC vesicle effectively protected the internal lipid bilayer structure and decreased the permeability of the lipid bilayer so that the drug could not easily leak. Moreover, the long‐term storage stability of the CC delivery system of the this vesicle is excellent; the particle size did not change considerably after storage for 3 months at 4 °C, and it exhibited a low drug leakage rate.

### 2.2 Plaque Targeting, Pharmacokinetic Properties, and Biodistribution of HA‐CC

**Figure 2:**
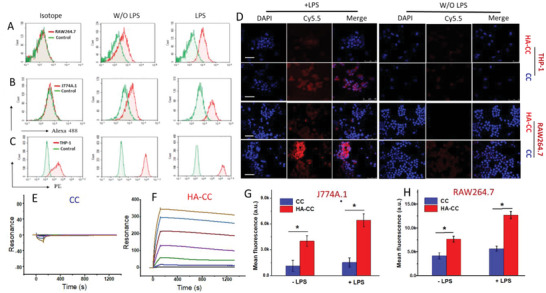
A–C) Flow cytometric analysis of CD44 expression in macrophages following LPS stimulation. D) Confocal laser scanning microscopy analysis of enhanced cellular internalization of HA‐CC in THP‐1 and RAW264.7 cells with or without 24 h LPS stimulation. Non‐targeted CC was the control. Scale bar: 50 µm. Improved hyaluronic acid‐modified cerasome (HA‐CC) macrophage interaction with or without 24 h LPS stimulation. SPR analysis of the binding between CD44 and CC F) with or E) without HA conjugation. Flow cytometric analysis of G) J774A.1 and H) RAW 264.7 cells after incubation for 2 h with fluorescent Cy5.5‐labeled HA‐CC (HA‐CC‐Cy5.5) or fluorescent Cy5.5‐labeled CC (CC‐Cy5.5). Differences among the groups were determined using ANOVA with Tukey's multiple comparison test (*n* = 5), and *p < 0.05 was considered significant.

Specific binding between CD44 and HA‐CC was demonstrated using the SPR analysis. As shown in **Figure** **2**E,F, HA‐CC bound strongly to CD44 on the SPR chip, but CC did not show any binding, demonstrating the role of the HA ligand. The cellular uptake of HA‐CC was measured in cultured macrophages of human and murine origin. Due to inflammatory signals inducing up‐regulation, a 2.1–2.8‐fold increase in CD44 expression after LPS stimulation was observed using flow cytometry (Figure 2A–C). Receptor‐mediated cellular uptake was evaluated using HA‐CC‐Cy5.5 and CLSM (Figure 2D). A 2 h incubation with HA‐CC produced an increase in cytosolic fluorescence intensity in LPS‐treated macrophages. In contrast, CC lacking HA modification exhibited a weak fluorescence signal, suggesting the HA dependence of CC uptake. Incubation of J774A.1 and RAW264.7 cells with HA‐CC produced a 3.9‐ and 2.05‐fold increase in cellular uptake compared to that of non‐targeted CC, as observed using quantitative flow cytometry analysis (Figure 2G,H). Therefore, it was concluded that the HA‐CD44 interaction resulted in the successful targeting of CC to activated macrophages.

**Figure 3:**
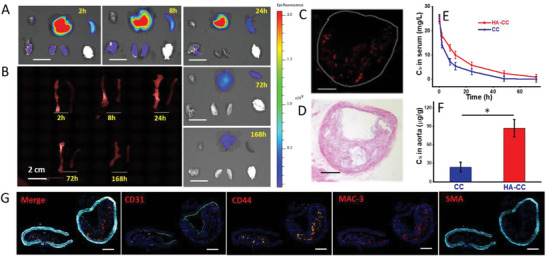
In vivo distribution and removal of fluorescent Cy5.5‐labeled hyaluronic acid‐modified cerasome (HA‐CC‐Cy5.5): ex vivo NIR images of A) the excised kidney, liver (Scale bar: 1.0 mm) and B) carotid artery after 2, 8, 24, 72, and 168 h. Preferential accumulation of HA‐CC on the plaque‐rich left carotid arteries. C) Accumulated HA‐CC‐Cy5.5 determined using fluorescence microscope. Lumen contour is indicated with a dashed white circle. Scale bar: 0.1 mm. D) Representative H&E staining image of the left carotid artery cross‐section. Scale bar: 0.1 mm. E) Pharmacokinetics of CC with or without HA modification (*n* = 3). F) Concentration levels of CC‐based nano‐carriers in aorta. Differences among the groups were determined using ANOVA with Tukey's multiple comparison test (*n* = 5). *p < 0.05 was considered significant. I) Fluorescence HIC analysis of CD44 overexpression in the plaque. Scale bar: 0.1 mm.

Immunohistochemical (IHC) analyses showed that CD44 was located in the plaque interior and was mainly overexpressed by macrophages (**Figure** **3**G). Thus, HA‐CC targeted active macrophages that overexpressed CD44 in the vulnerable plaques. Apoe^−/−^ mice were administered with fluorophore‐labeled HA‐CC‐Cy5.5 at different time intervals, and in vivo targeting and enrichment of HA‐CC in atherosclerotic plaques were evaluated. As shown in Figure 3B, the plaque‐rich left carotid and control right carotid arteries were examined by monitoring fluorescence enhancement. Bright fluorescence was observed in the left carotid artery at 2 h post‐injection, with maximum fluorescence at 24 h post‐injection, and detectable fluorescence remained up to 7 d after administration. The same accumulation of HA‐CC‐Cy5.5 was not observed in the right carotid artery, indicating in vivo targeting. Histological examination of the left carotid arteries confirmed the colocalization of the fluorescence signal and atherosclerotic plaques (Figure 3C,D), further indicating the enrichment of HA‐CC in the plaques. The HA‐CC‐Cy5.5 was also found in the liver and kidney, consistent with the known distribution patterns of nano‐formulations (Figure 3A), and gradually filtered away, as indicated by the radiant efficiency of liver from nearly 2 × 10^9^ at the first 2 h to nearly 0.4 × 10^9^ after 168 h. A pharmacodynamic investigation of the circulation stability and targeting capacity of HA‐CC, which is considered to be partly determined by surface properties, was conducted. The presence of HA prolonged the CC circulation time, favoring HA‐CC enrichment in vulnerable plaques (Figure 3E). Monitoring Si concentrations in plaques revealed preferential accumulation of HA‐CC over CC (Figure 3F).

### 2.3 Screening Vulnerable Plaques Using HA‐CC‐Gd Mediated MRI Tool

**Figure 4:**
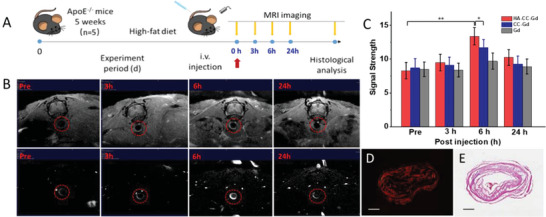
Accumulation and imaging of hyaluronic acid‐modified cerasome gadodiamide (HA‐CC‐Gd) in moderate aorta atherosclerotic models. A) Schematic illustration of the MRI of aorta plaque in vivo. B) MRI of atherosclerotic aorta at different time points (pre‐injection; 3, 6, and 24 h post‐injection). The vessel wall is indicated with a red circle, and plaque is indicated with a red circle. MRI signals of plaques were enhanced by rapid acquisition with a relaxation enhancement (RARE) sequence at T1 weighted image. C) Quantification of MRI signal strength after addition of different MRI agents. Differences among the groups were determined using ANOVA with Tukey's multiple comparison test (*n* = 5). *p < 0.05 was considered significant, and **p < 0.01 was considered highly significant. D) Fluorescence image and E) histology of plaques after MRI testing. Adjacent tissue cross‐sections were monitoring fluorescence imaging and then stained with H&E.

MRI offers submillimeter level resolution with unlimited penetration depth; hence, it is a widely used noninvasive technique for the diagnosis of atherosclerotic lesions.^[^ ^18^, ^19^ ^]^ Apoe^−/−^ mice were intravenously injected with CC‐Gd with or without HA modification, as well as free Gd, and then imaging was performed before and after injection at 3, 6, and 24 h (**Figure** **4**A). A strong MRI signal was observed in the atherosclerotic plaque at 3 h post‐injection and lasted for 24 h before returning to pre‐injection values, indicating HA‐CC‐Gd clearance (Figure 4B). Dose of 0.5 mg kg^−1^ HA‐CC‐Gd increased the MRI signal strength by approximately 80% and 40% at 6 h (Figure 4C) compared to that of the non‐targeted CC‐Gd and free drug, suggesting preferential accumulation. Cy5.5‐labeled HA‐CC‐Gd was also administered to Apoe^−/−^ mice for a dual‐mode location to ensure the MRI signal was from HA‐CC‐Gd. Mice were sacrificed after MRI, and histological examination of aortas revealing results similar to those of pathological, fluorescent, and MRI images suggested outstanding in vivo vulnerable plaque recognition by HA‐CC (Figure 4D,E).

### 2.4 Plaque Regression Effect of HA‐CC–RST

**Figure 5:**
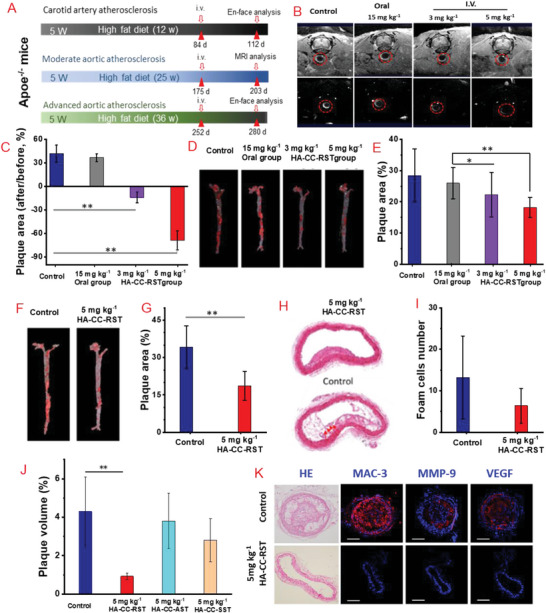
In vivo efficacy of hyaluronic acid‐modified cerasome rosuvastatin (HA‐CC–RST) in reversing plaques of differing severity in different Apoe^−/−^ mice models. A) Schematic illustration of anti‐atherosclerosis therapy in vivo. B) MRI of aorta plaque at 6 h post‐treatment HA‐CC‐Gd for advanced atherosclerosis model at the end of treatment. C) Plaque area measurements for moderate aortic atherosclerosis. D) Oil Red O stain of aortas moderate atherosclerosis. E) Quantification of plaque area, moderate atherosclerosis. F) Oil Red O stain of aortas, severe atherosclerosis. G) Quantification of plaque area, advanced atherosclerosis. H) H&E stained cross‐sections of aortas, advanced atherosclerosis. I) Foam cell numbers determined by histology analysis in advanced atherosclerosis. J) Quantification of plaque volume. K) Histological cross sections: H&E, immunohistochemical staining with antibodies against MAC‐3, MMP‐9, and VEGF of left carotid arteries; placebo and HA‐CC–RST 5 mg kg^−1^ group. Differences among the groups were determined using ANOVA with Tukey's multiple comparison test (*n* = 5). *p < 0.05 was considered significant, and **p < 0.01 was considered highly significant.

Atherosclerosis is a complex chronic inflammatory disease,^[^ ^20^ ^]^ and targeted treatment of inflammation is an attractive therapeutic approach.^[^ ^21^, ^22^ ^]^ Oral statins are the first drug of choice for the disease and are widely prescribed to inhibit cholesterol synthesis and reduce cholesterol levels.^[^ ^23^ ^]^ Statins have pleiotropic effects, including anti‐inflammatory effects, enhanced macrophage phagocytosis, and improved macrophage efferocytosis^[^ ^24^, ^25^, ^26^ ^]^ Potent immunomodulatory effects have been ascribed to hydroxymethylglutaryl‐coenzyme A reductase inhibition in inflammatory cells^[^ ^27^, ^28^, ^29^ ^]^ and RST has been found to exhibit higher lipid‐lowering effect than other statins. However, a recent meta‐analysis demonstrated that the anti‐atherosclerotic effects of RST were independent of its lipid‐lowering effect. Thus, targeted delivery of RST to plaques will probably result in increased plaque regression.^[^ ^23^ ^]^ The effects of HA‐CC–RST were investigated in three different models, carotid artery atherosclerosis, as well as moderate and severe aortic atherosclerosis, established by adjusting the duration of high‐fat feeding (**Figure** **5**A). For carotid artery atherosclerosis, the mean plaque area in HA‐CC–RST‐treated mice was significantly lower than that in the oral statin‐treated or placebo mice. Carotid artery atherosclerosis mice administered a high‐dose of HA‐CC–RST showed a 79% decrease in plaque area compared to that in oral statin‐treated or placebo mice (Figure 5C). For moderate aorta atherosclerotic models, Oil red O staining of lipid‐rich plaques showed a 13.3% decrease in plaque area in the low dose HA‐CC–RST‐treated group and a 29.5% decrease in the high dose treated group compared to that in placebo or oral statin‐treated groups (Figure 5D,E). MRI with HA‐CC–Gd also demonstrated plaque regression (Figure 5B). In advanced aorta atherosclerotic models, high‐dose HA‐CC–RST treatment produced a 56.3% decrease in plaque area, suggesting that RST delivered by HA‐CC is primarily responsible for the anti‐atherosclerosis effect (Figure 5F,G). Histological analysis showed a significant decrease in plaque area in the high‐dose HA‐CC–RST group compared to that in the placebo group (Figure 5H). The number of foam cells was also markedly decreased in the plaques, demonstrating a remarkable regression of the plaques (Figure 5I). Comparing different statin‐based actively targeted HA‐CC, RST nano‐agent demonstrated the most prominent plaque regression, achieving a 78.7% decrease in plaque volume (Figure 5I). Histological analysis and immunostaining showed increased number of cholesterol crystal‐rich atherosclerotic plaques and enhanced expression of many inflammatory factors, such as MAC‐3, MMP‐9, and VEGF, in the left carotid artery in the placebo group (Figure 5K). In contrast, the high dose 5 mg kg^−1^ HA‐CC–RST‐treated group showed reduced expression of inflammatory markers. Moreover, HA‐CC–RST was more effective in atherosclerotic regression than the systemic administration of RST, supporting the notion that targeted delivery of RST to the plaque site to inhibit local inflammation is a viable approach against atherosclerosis.

### 2.5 Pharmacological Effect of HA‐CC–RST in Plaque Regression

**Figure 6:**
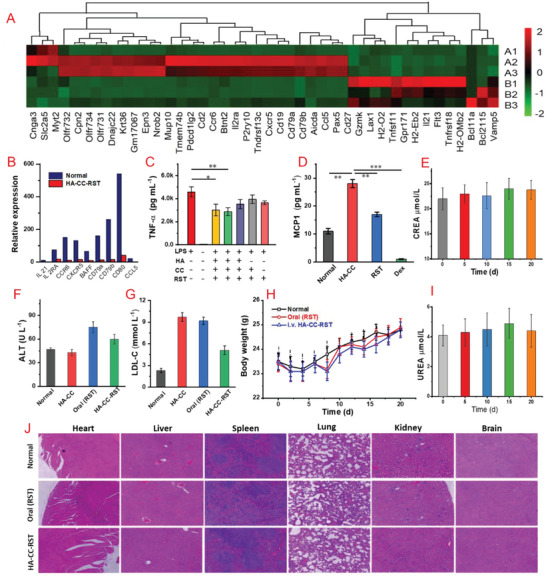
A) RNA‐Seq transcriptomic analysis: differentially expressed genes (DEGs) in the hyaluronic acid‐modified cerasome rosuvastatin (HA‐CC–RST) treatment group. B) Representative down‐regulated genes. Intravenous injection of HA‐CC was used as control. C) The release of TNF‐*α* from after treating THP1 with different agents. D) RST reduced the production of MCP1 by human macrophages. THP1 monocytes were differentiated with 100 nm PMA for 3 days and treated with 10 um RST or Dexamethasone (Dex) for 24 h. Quantification of F) ALT and G) LDL‐C concentrations in plasma (Oral RST: 5.0 mg mL^−1^; HA‐CC–RST: 5 mg mL^−1^). H) Body weight changes of mice during the treatment period. E,I) UREA and CREA concentration monitoring during treatment. J) H&E results for main organs. Differences among the groups were determined using ANOVA with Tukey's multiple comparison test (*n* = 5). *p < 0.05 was considered significant, and **p < 0.01, ***p < 0.01 were considered highly significant.

Macrophages are key components of atherosclerotic initiation, progression, and regression and promote the local inflammatory response by secreting pro‐inflammatory cytokines, chemokines, and reactive oxygen and nitrogen species. The anti‐inflammatory effect of RST was demonstrated by its inhibition of TNF‐*α* and MCP‐1 secretion in THP‐1 cells (**Figure** **6**C,D). To further understand the plaque regression effect, RNA‐Seq transcriptomic studies following 5 mg kg^−1^ HA‐CC–RST treatment every alternate day for 3 days were performed on the severe aortic atherosclerosis model; the mice were sacrificed on day 4, and aortas were isolated. HA‐CC–RST treatment suppressed the expression of a cluster of inflammatory genes (CCL5, CCR6, and CXCR5) as well as multiple chemokine‐related genes (CD79a, CD79b) (Figure 6A).

In contrast, LDL‐C levels were not significantly lower in the HA‐CC–RST group than those in the control group, suggesting that the anti‐atherosclerotic effects were not exerted via lipid‐lowering (Figure 6G). Therefore, HA‐CC–RST might downregulate the expression of inflammatory and chemokine‐related genes, thus inhibiting the migration, activation, infiltration, and proliferation of macrophages in plaques, decreasing the number of foam cells, and reducing plaque inflammation.

In terms of biosafety, alanine aminotransferase levels were higher following RST treatment than those following HA‐CC or 5 mg kg^−1^ HA‐CC–RST treatment (Figure 6F), suggesting that HA‐CC–RST may have reduced deleterious effects on liver when compared to RST. Both CREA and UREA remained stable during treatment, indicating that HA‐CC–RST did not cause obvious renal injury (Figure 6E,I). The body weights of the mice also increased during treatment (Figure 6H). H&E staining indicated no distinct organ injuries (Figure 6J). These results demonstrated the safety of HA‐CC‐mediated RST delivery. In summary, a new targeting agent with enhanced feasibility, effectiveness, and safety for the treatment of atherosclerotic plaques has been developed.

## 3 Conclusion

The HA‐guided CC for targeted delivery of diagnostic and therapeutic agents into atherosclerotic plaques has been developed successfully. The high affinity between the HA ligand and plaque surface CD44 conferred improved targeting capacity. Drugs were able to overcome the mass transfer barrier and were delivered to lipid‐rich hydrophobic atherosclerotic plaques. Increased drug delivery efficiency was evident from the requirement of 50‐fold lower doses of HA‐CC–RST than those of oral RST, significantly reversed atherosclerosis, 56.3% and 78.7% reduction in plaque area and volume, respectively. The results were further corroborated by the RNA‐Seq transcriptomic analysis that revealed the pleiotropic effects of RST during plaque regression. Furthermore, the drug delivery system can be used to transport any payload into atherosclerotic plaques. Thus, HA‐CC is anticipated to be a multifunctional platform for the therapy of vulnerable plaques and the early diagnosis of plaques with a high risk of MACEs. This platform has promising potential for reducing mortality caused by atherosclerosis.

## 4 Experimental Section

### Materials and Instruments

Sodium hyaluronate (HA) with an average molecular weight of 10 kDa was purchased from Huaxi Biopharmaceutical Co., Ltd. (Sichuan, China); mPEG2000‐DSPE‐Cy5.5, phosphatidylethanolamine (DMPE), and distearoyl phosphatidylcholine (DSPC) from Corden Pharma Switzerland LLC (Switzerland); cholesterol (Chol), *N*‐hydroxysulfosuccinimide sodium salt (NHSS), 1‐ethyl‐3‐(3‐dimethylaminopropyl) carbodiimide hydrochloride (EDC), rosuvastatin (RST), atorvastatin (AST), simvastatin (SST), and Gadodiamide (Gd) were purchased from Sigma‐Aldrich Chemical Co. (St. Louis, MO, USA). Anti‐vascular endothelial growth factor (VEGF) and anti‐matrix metallopeptidase‐9 (MMP‐9) monoclonal antibodies were purchased from Cell Signaling Technology (Danvers, MA, USA). Rabbit anti‐CD44, anti‐macrophage inflammatory protein 3 alpha (MAC‐3), rabbit recombinant anti‐CD31 polyclonal, and rabbit anti‐alpha smooth muscle actin (anti‐SMA) monoclonal antibodies were purchased from Abcam (Shanghai, China). Human recombinant CD44 protein with an hFc tag was purchased from Sino Biological Inc (Beijing, China).

### Animal Models

For demonstration the efficiency of HA‐CC‐RST for atherosclerosis with different severity, different the SPF‐grade Apoe^−/‐^ mice (6 weeks old; weight, 16 ± 1 g) were obtained from Beijing Vital River Laboratory Animal Technology Co., Ltd. (Beijing, China) and maintained at 22 °C with a 12‐h light/dark cycle and food and water was provided ad libitum. Three types of plaque‐bearing animal models, that is, the moderate, advanced aorta, and left carotid atherosclerosis model, were established as follows: Apoe^−/−^ mice on a high‐fat diet were fed rodent chow until 8 weeks of age, after which an atherogenic diet containing 10% fat and 2% cholesterol was administered for 24 and 36 weeks for obtaining the moderate and advanced atherosclerosis models, respectively; the left carotid atherosclerosis model was established by implanting a cannula in the left carotid artery of the Apoe^−/−^ mice to cause carotid artery stenosis, followed by high‐fat diet feeding for 12 weeks. All animal studies were performed in accordance with the animal welfare requirements defined by the Laboratory Animals Welfare Office of the Capital Medical University.

### Preparation of the HA‐Functionalized CC by Thin‐Film Hydration

Organoalkoxysilane lipid 1 (CL‐1) was synthesized as previously described. CCs containing CL‐1, DSPC, and DMPE at a mass ratio of 5:3:1, and control liposomes (LPs) containing DSPC, cholesterol, and DMPE at a molar ratio of 5:3:1 were selected. To compare the therapeutic effects, RST, AST, and SST were encapsulated in the lipid bilayer of CCs with a drug to lipid molar ratio of 1:10. In brief, lipids and different statins were dissolved in ethanol simultaneously, evaporated to dryness in a rotary evaporator, hydrated at 37 °C for 1 h in ultrapure water, and then the resulting emulsion was processed using probe sonicator at 180 W for 10 min. Fluorophore‐labeled CC was prepared by adding mPEG2000‐DSPE‐Cy5.5 (1% of total lipids) to the CC formulation, according to the aforementioned protocol. Hydrophilic Gd was encapsulated using a Gd aqueous solution (1.0 mg mL^−1^) as the hydrating solution after rotary evaporation using the aforementioned procedure. The procedures for the preparation of control liposomes are provided in the Supporting Information.

Activation of the HA carboxyl group was required for HA modification of CC. HA was dissolved in aqueous solution of NHSS and EDC for 1 h at 37 °C, and the activated HA was separated by adding 10 volumes of ethanol followed by centrifugation. The active HA ester was dissolved in 10 mm NaHCO_3_ buffer to obtain a final pH of pH 8.5 and incubated with CC or LP overnight at room temperature prior to purification of HA‐modified nanovesicles using ultrafiltration centrifugation (50 KD).

### Characterization of Nano‐Formulations

Nano‐formulations were diluted 100‐fold in ultrapure water, and the size distribution and zeta potential were characterized using a Zetasizer Nano ZS90 (Malvern, UK). The morphology was analyzed using a transmission electron microscope (HT7800, HITACHI, Japan). The encapsulation efficiency (EE) and drug release testing procedures are described in the Supporting Information. In brief, free drug was recovered by ultrafiltration centrifugation, and nano‐formulations were disrupted and diluted with methanol to release the encapsulated drug for measurement. The RST concentration was determined using HPLC, and Gd and CC contents were determined using inductively coupled plasma–mass spectrometry (ICP–MS) to assess Gd and Si elements. The EE was calculated using the following formula:

(1) $$\mathrm{EE}=C_{\mathrm{free}}/C_{\mathrm{total}}\times 100\%$$

### In Vitro Targeting Capacity Validation

For achieving SPR data, the Biacore T200 (Cytiva, USA) was used to monitor CD44 and HA‐CC binding using a Biacore sensor chip (Protein A). A 10 mm glycine‐HCl (pH 1.5) buffer solution was used for chip regeneration, and 1× PBS aqueous solution (pH 7.4) was used as the running buffer.

RAW264.7, J774A.1, and THP‐1 cells were cultured in 100 × 20 mm dishes containing Dulbecco's modified Eagle's medium supplemented with 10% fetal bovine serum, penicillin (100 U mL^−1^), streptomycin (0.1 mg mL^−1^), nystatin (12.5 U mL^−1^), and l‐glutamine (2 mm). The cells were harvested, washed twice with PBS, centrifuged, and resuspended in PBS (0.5 mL). Flow cytometry was performed using Alexa Fluor 488 rat anti‐human CD44 and IgG2b isotype control antibodies on a flow cytometer (BD FACS Calibur, USA) at the FL2‐channel (excitation: 488 nm, emission: 560 nm), and 1 × 10^4^ events were analyzed per sample.

The endocytic capacity of THP‐1 and RAW264.7 cells with or without 24‐h LPS stimulation was assessed by confocal laser scanning microscopy (CLSM). THP‐1 and RAW264.7 were seeded into a 6‐well plate at a density of 5 × 10^5^ cells/well and incubated with fluorescent Cy5.5‐labeled HA‐CC (HA‐CC‐Cy5.5) or fluorescent Cy5.5‐labeled CC (CC‐Cy5.5) at 37 °C for 2 h. The cells were fixed with 4% paraformaldehyde solution and nuclei stained with DAPI for 10 min before CLSM observation. The HA inhibition was evaluated by adding HA to the culture medium after PBS washing followed by CLSM. Enhanced HA‐CC interaction with J774A.1 and RAW264.7 cells was determined by flow cytometry after further incubating for 2 h with HA‐CC‐Cy5.5. Fluorescence intensity allowed comparison of endocytosis with and without 24 h LPS stimulation.

### Pharmacokinetics, Plaque Targeting, and Tissue Distribution

The non‐targeted CC‐Cy5.5 and HA‐CC‐Cy5.5 (0.1 mg Si kg^−1^) were administered via the mouse tail vein, and organ distribution at 2 h, 8 h, 24 h, 48 h, 72 h, and 7 days HA‐CC‐Cy5.5 was visualized using an in vivo imaging system (CRI Maestro 2, USA).

The Apoe^−/−^ mice were fed a high‐fat diet for at least 36 weeks and were randomly divided into 2 groups of 5 according to body weight and were intravenously administered a single large dose of CC (0.1 mg Si kg^−1^) with or without modifying HA. Mice were sacrificed 2 h post treatment, and aortas were dissected for measuring nanovesicle concentration based on silicon content using ICP–MS to assess plaque targeting.

SD rats (Beijing Vital River Laboratory Animal Technology Co., Ltd.) of mixed gender were randomly divided into 2 groups of 5 and were intravenously administered a single large dose of 0.1 mg Si kg^−1^ of CC with or without modifying HA. Blood samples were collected at 0, 1, 2, 6, 8, and 24 h post administration, centrifuged, and CC concentration with or without HA ligand calibrated by Si concentration was measured using ICP–MS to evaluate pharmacokinetic properties.

### In Vivo MRI Screening of Plaques in Apoe^−/−^ Mice

Images were obtained using a 9.4 T (Bruker 94/30 USR) small animal magnet with a birdcage coil having a diameter of 30 cm at Tsinghua University. Mice were anesthetized with 1.5% isoflurane air mixture at 35–37 °C with warm air flowing through the bore, and respiration was monitored (MP150, Biopac, Goleta, CA). Pre‐injection images were captured 1 day prior to treatment. CC–Gd, with or without HA modification, was injected via the tail vein, and control mice were was administered with Gd solution. The imaging was performed prior to the injection and at 3, 6, and 24 h post‐injection. The imaging sequences used were 1_Localizer, TOF_2D_FLASH, and T1_FLASH. The detailed MRI parameter settings are provided in the Supporting Information. The signal strength of the transverse section of the aortic plaque was obtained using Bruker Paravision 360 2.0, and MRIcron (Version 11).

### HA‐CC‐RST Therapy of Apoe^−/−^ Mice

Apoe^−/−^ mice fed a high‐fat diet for 24 weeks were randomly divided into four groups according to body weight and plasma LDL‐cholesterol (LDL‐C) levels. Mice were treated with vehicle (5% glucose) or administered 1.25, 2.5, or 5 mg RST kg^−1^ of HA‐CC–RST via the tail vein once daily for 28 days. Body weight was measured once or twice weekly. Dissection was performed from the proximal ascending aorta to the bifurcation of the iliac artery using a dissecting microscope. The aorta was opened longitudinally, pinned flat onto black dissecting wax, stained with Oil red O, and photographed at fixed magnification. The percentage of the total aortic area containing lesions was calculated to evaluate plaque progression.

### Transcriptomic Analysis of Atherosclerotic Aortas

Apoe^−/−^ mice fed with a high‐fat diet for 24 weeks were randomly divided into two groups according to body weight and plasma LDL‐C levels and administered 5 mg kg^−1^ HA‐CC–RST via the tail vein once daily for 14 days. Mice were sacrificed on day 29 and aortas were analyzed. A total of 1 µg RNA per sample was used for sequencing, and libraries were generated using the NEBNext UltraTM RNA Library Prep Kit for Illumina (NEB, USA), according to the manufacturer's protocol, and index codes were added to attribute sequences of each sample. The experimental details are provided in Supporting Information. THP‐1 monocytes were differentiated with 100 nm PMA for 3 days and treated with 10 *µ* m RST or 0.1 *µ* m dexamethasone for 24 h. MCP1 levels in the media were measured using the MCP1 ELISA kit (Invitrogen).

### Statistical Analysis

All data were presented as the means ± standard deviations (SD) from at least three repeated experiments. Differences among the groups were determined using one‐way analysis of variance (ANOVA) with Tukey's multiple comparison test. Sample size (*n*) for each statistical analysis was not less than 5. Kaplan–Meier survival curves were generated using the log‐rank test for comparisons using Prism 7. *p < 0.05 was considered significant, and **p < 0.01 was considered highly significant.

## Conflict of Interest

The authors declare no conflict of interest.

## Supporting information

Supporting Information

## Data Availability

The data that support the findings of this study are available from the corresponding author upon reasonable request.
