# Supplementary material for: Hyaluronic Acid‐Guided Cerasome Nano‐Agents for Simultaneous Imaging and Treatment of Advanced Atherosclerosis
Source: Adv Sci (Weinh). 2022 Dec 18;10(5):2202416. doi: 10.1002/advs.202202416 (PMC9929131; doi:10.1002/advs.202202416)
Supplement: Supplementary file 1 — Supporting Information [file ADVS-10-2202416-s001.pdf]

ADVANCED SCIENCE
Open Access

Supporting Information

for *Adv. Sci.*, DOI 10.1002/advs.202202416

Hyaluronic Acid-Guided Cerasome Nano-Agents for Simultaneous Imaging and Treatment of Advanced Atherosclerosis

Qian Ma, Sijing Wu, Ling Yang, Yaohua Wei, Chaoyong He, Wenshan Wang, Yingxin Zhao, Zhijian Wang, Shiwei Yang, Dongmei Shi, Yuyang Liu, Zhiming Zhou, Jiefang Sun and Yujie Zhou**

Hyaluronic Acid-Guided Cerasome Nano-Agents for Simultaneous Imaging and Treatment of Advanced Atherosclerosis

Qian Ma[1,3,4], Sijing Wu[1,3], Ling Yang[4], Yaohua Wei[4], Chaoyong He[4], Wenshan Wang[4], Yingxin Zhao[1,2,3], Zhijian Wang [1,2,3], Shiwei Yang[1,2,3], Dongmei Shi[1,2,3], Yuyang Liu[1,2,3], Zhiming Zhou[1,2,3], Jiefang Sun[4]* and Yujie Zhou [1,2,3]*

[1] Department of Cardiology, Beijing Anzhen Hospital, Capital Medical University

[2] Beijing Institute of Heart Lung and Blood Vessel Disease

[3] Beijing Key Laboratory of Precision Medicine of Coronary Atherosclerotic Disease, Clinical Center for Coronary Heart Disease, Capital Medical University, Beijing 100029, China

[4] Beijing Inno Medicine Co., Ltd, Beijing 100195, China

*Correspondence to: Prof. Dr. Yujie Zhou, Department of Cardiology, Beijing Anzhen Hospital, Capital Medical University, Beijing 100029, China (Tel: 86-10-64456489. Fax: 86-10-64442234. Email: azzyj12@163.com)

Dr. Jiefang Sun, Beijing Inno Medicine Co., Ltd, Beijing 100195, China (Tel: 86-10-84362596. Email: sunjf2001@163.com)

1. Experiment

1.1 Left carotid artery cannula implantation for constructing the left carotid atherosclerosis model

The mice were anaesthetized by intraperitoneal injection of 1% sodium pentobarbital (prepared by adding 1 mg of sodium pentobarbital to 100 ml of normal saline) at a dose of 40 mg/kg. Then, the mice were fixed on the surgical plate in the supine position, disinfected around the neck with 75% (v/v) alcohol, the neck skin was cut longitudinally, the anterior cervical gland was bluntly separated, and the beating left common carotid artery can be observed on the left side of the trachea. The common carotid artery was carefully separate to the bifurcation. A silicone cannula with a length of 2.5 mm and an inner size of 0.3 mm was placed on the outer periphery of the left common carotid artery. The proximal and distal segments of the cannula were narrowed and fixed by filaments. Local tightening causes rapid blood flow in the proximal end with increased shear force, and thus damage to the intima of the blood vessel. The carotid artery was repositioned, and the neck skin was intermittently sutured. All operations were performed under a 10x stereomicroscope.

1.2 Preparation of CC based nanoformulations

After dissolved all the chemicals and mixed, the organic solvent was completely removed through rotary evaporation (55 °C water bath, 90 r min-1, 30 min), and leaving a film on the wall of the vessel. Millipore water was added into the flask to reach a final concentration of 1 mg ml^{-1}, and the film was placed in a 50 °C water bath for 30 min, which allows the full hydration of the film. This is followed by

ultrasonication for 10 min in a water bath and ultrasonically probed for 5 min (amplitude 20, interval 3 s). The solution was placed in a 50 °C oven to promote the sol-gel process, and finally, the siliceous drug-loaded nanoparticles were purified by removing the excess drug by gel column chromatography, and the amount of RST loaded into the CC-RST was calculated by the difference in added and removed amount. For achieving atorvastatin or simvastatin encapsulated CC, i.e. CC-AST or CC-ST, similar preparing methods were used, excepting substituting RST with AST/ST. The drug to lipid ratios remained unchanged for all.

For conjugating hyaluronic acid (HA), 50 mg of HA (MW 10kD) was dissolved in 10 mL D.I. water, and then 12 mg EDC and 14 mg sulfo-NHS were added in the HA solution. The active reaction was processed in room temperature for 2 h. Subsequently, 90 mL ethanol was poured into the solution to precipitate HA. Finally, the gel-like sulfo-NHS-HA was collected by centrifugation at 8000 rpm for 5min. After removing the supernatant, the sulfo-NHS-HA was re-dissolved in 1mL DI water. The resulted sulfo-NHS-HA was mixed with the CC, and incubated overnight under magnetic stirring in a 4 °C refrigerator. Finally, the excess sulfo-NHS-HA was removed by gel column chromatography to obtain the drug-loaded cerasomes, i.e. HA-CC-D.

1.0 mL of HA-CC-D suspension was taken, and the suspension was allowed to form a strong acidic environment by adding excess amount of HCl. The suspension was further ultrasonicated to accelerate the release of the drug from the HA-CC-D. The drug content in the resulting liquid was measured by HPLC (Waters 2487, Waters

Corporation, U.S.A.), and the encapsulation rate was calculated in accordance with Equation 1.

1.3 Determination of drug-loading rate

The method for determining the drug-loading rate is similar to that for determining the encapsulation rate, except that the calculation method is slightly different. HA-CC-D suspension was taken, and the suspension was allowed to form a strong acidic environment by adding excess amount of HCl. The suspension was further ultrasonicated to accelerate the release of the drug from the HA-CC-D. The drug content in the resulting liquid was measured by HPLC (Waters 2487, Waters Corporation, U.S.A.), and the drug-loading rate was calculated in accordance with Equation 2.

1.4 Investigation of long-term encapsulation rate

The CC-R and the CC-Ga were stored at 4℃, and sampled at different time points, and the free drug was removed by ultrafiltration and centrifugation to detect changes in the encapsulation rate thereof, and the results are shown in Figure 1. As shown in the figure, there were no significant changes in the drug contents in the cerasome delivery systems CC-R and the CC-Ga after 90 days of standing. This indicates that the inorganic polysiloxane network on the surface of the CC vesicle effectively protects the internal lipid bilayer structure and decreases the permeability of the lipid bilayer, so that the drug is not easily leaked. The data above clearly demonstrats that the long-term storage stability of the cerasome delivery system of the present disclosure is excellent, and the particle size does not change much after storage for

three months at 4°C with low leakage rate of the drug.

At 2 h, 8 h, 24 h, 48 h, 72 h and 7 d after HA-CC-Cy5.5 injection, the mice were immediately sacrificed, and the intact major organs (including heart, liver, spleen, lung, and kidney) were collected and analyzed using the vivo fluorescence imaging systems (CRI Maestro 2, USA) to display the distribution of HA-CC-Cy5.5 in various organs.

For in vivo plaque targeting analysis, totally 18 male Apoe$^{-/-}$ mice fed with a high-fat diet for at least 40 weeks were randomly divided into 4 groups according to their body weight: CC with or without HA modification, with 3-5 mice in each group. The animals were administered by single intravenous bolus injection. The mice were euthanatized after 2h post dose, and the aortas plaque of each group were collected for determining the concentration of according to Si concentrations using ICP-MS.

For pharmacokinetic analysis, thirty-six SD rats (half male and half female) were randomly divided into 3 groups (3 rats in each group/sex) and were given 50 mg/kg (Si concentration) of CC with/without modifying HA, respectively by single intravenous bolus injection, and the dose volume was 20 mL/kg. Blood samples were collected in EDTA-K_2 coated centrifuge tube at pre-dose, 0 (±1 min), 1 h, 2 h, 6 h, 8 h and 24 h after administration. The blood samples were centrifused to obtain the plasma samples for the determination of free drug and total rosuvastatin concentration. The ICP-MS method was used to determine the concentration of HA-CC in the plasma.

1.5 Transcriptome analysis from aortas of murine atherosclerosis

1.5.1 Library preparation for Transcriptome sequencing

A total amount of 1 μg RNA per sample was used as input material for the

RNAsample preparations. Sequencing libraries were generated using NEBNext® UltraTM RNA Library Prep Kit for Illumina® (NEB, USA) following manufacturer's recommendations and index codes were added to attribute sequences to each sample. Briefly, mRNA was purified from total RNA using poly-T oligo-attached magnetic beads. Fragmentation was carried out using divalent cations under elevated temperature in NEBNext First Strand Synthesis Reaction Buffer (5X). First strand cDNA was synthesized using random hexamer primer and M-MuLV Reverse Transcriptase (RNase H-). Second strand cDNA synthesis was subsequently performed using DNA Polymerase I and RNase H. Remaining overhangs were converted into blunt ends via exonuclease/polymerase activities. After adenylation of 3' ends of DNA fragments, NEBNext Adaptor with hairpin loop structure were ligated to prepare for hybridization. In order to select cDNA fragments of preferentially 250~300 bp in length, the library fragments were purified with AMPure XP system (Beckman Coulter, Beverly, USA). Then 3 μl USER Enzyme (NEB, USA) was used with size-selected, adaptor-ligated cDNA at 37°C for 15 m in followed by 5 min at 95 °C before PCR. Then PCR was performed with Phusion High -Fidelity DNA polymerase, Universal PCR primers and Index (X) Primer. At last, PCR products were purified (AMPure XP system) and library quality was assessed on the Agilent Bioanalyzer 2100 system.

1.5.2 cluster generation

The clustering of the index-coded samples was performed on a cBot Cluster Generation System using TruSeq PE Cluster Kit v3-cBot-HS (Illumia) according to

the manufacturer's instructions. After cluster generation, the library preparations were sequenced on an Illumina Novaseq platform and 150 bp paired-end reads were generated.

1.5.3 Quality control

Raw data (raw reads) of fastq format were firstly processed through in-house perl scripts. In this step, clean data (clean reads) were obtained by removing reads containing adapter, reads containing ploy-N and low quality reads from raw data. At the same time, Q20, Q30 and GC content the clean data were calculated. All the downstream analyses were based on the clean data with high quality.

1.5.4 Reads mapping to the reference genome

Reference genome and gene model annotation files were downloaded from genome website directly. Index of the reference genome was built using Hisat2 v2.0.5 and paired-end clean reads were aligned to the reference genome using Hisat2 v2.0.5.

We selected Hisat2 as the mapping tool for that Hisat2 can generate a database of splice junctions based on the gene model annotation file and thus a better mapping result than other non-splice mapping tools.

1.5.5 Quantification of gene expression level

Counts v1.5.0-p3 was used to count the reads numbers mapped to each gene. And then FPKM of each gene was calculated based on the length of the gene and reads count mapped to this gene. FPKM, expected number of Fragments Per Kilobase of transcript sequence per Millions base pairs sequenced, considers the effect of sequencing depth and gene length for the reads count at the same time, and is

currently the most commonly used method for estimating gene expression levels.

1.5.6 Differential expression analysis

Differential expression analysis of two conditions/groups (two biological replicates per condition) was performed using the DESeq2 R package (1.16.1). DESeq2 provide statistical routines for determining differential expression in digital gene expression data using a model based on the negative binomial distribution. The resulting P-values were adjusted using the Benjamini and Hochberg's approach for controlling the false discovery rate . Genes with an adjusted P-value <0.05 found by DESeq2 were assigned as differentially expressed.

1.5.7 GO and KEGG enrichment analysis of differentially expressed genes

Gene Ontology (GO) enrichment analysis of differentially expressed genes was implemented by the clusterProfiler R package, in which gene length bias wascorrected.GO terms with corrected Pvalue less than 0.05 were considered significantly enriched by differential expressed genes.

KEGG is a database resource for understanding high-level functions and utilities of the biological system, such as the cell, the organism and the ecosystem, from molecular-level information, especially large-scale molecular datasets generated by genome sequencing and other high-through put experimental technologies (http://www.genome.jp/kegg/). We used clusterProfiler R package to test the statistical enrichment of differential expression genes in KEGG pathways.

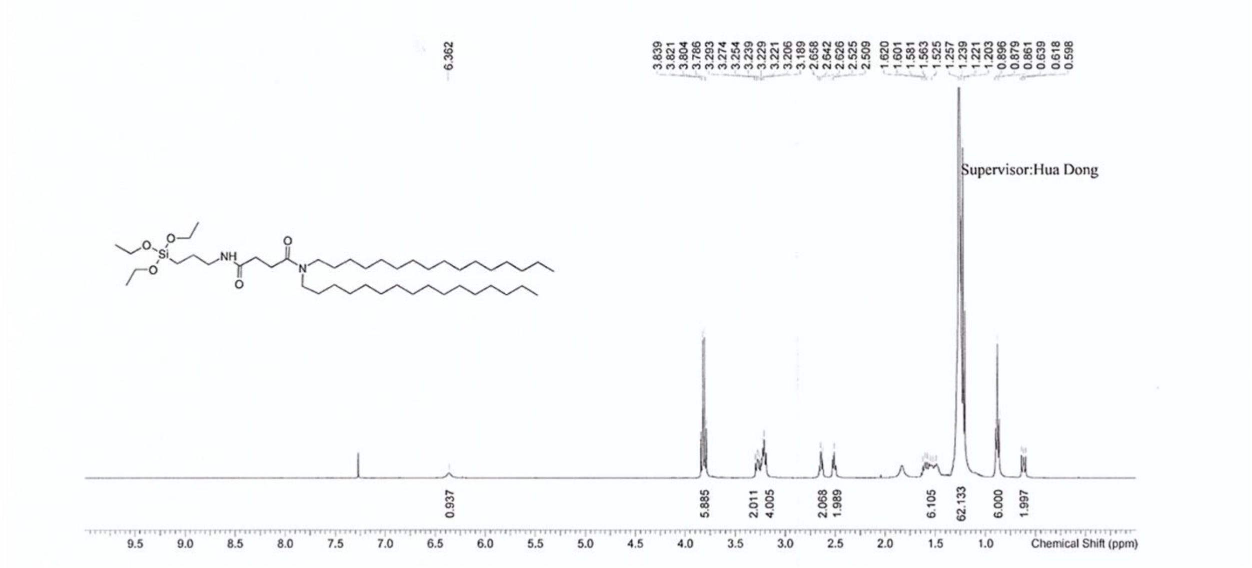

Figure S1. The chemical structure of organoalkoxysilane amphiphile (CL-1) and its ^{1}H NMR result. ^{1}H NMR (400 MHz, CDCl3, 25℃, MS) : d=0.59 (t, J=8.4 Hz, 2H, $SiCH_2$), 0.85 (t, J=6.6 Hz, 6H, $NCH_2CH_2(CH_2)_{13}CH_3$), 1.20 (t, J=7.3 Hz, 9H, $SiOCH_2CH_3$), 1.22~1.25 (m, 52H, $NCH_2CH_2(CH_2)_{13}CH_3$), 1.60 (br, 6H, $NCH_2CH_2(CH_2)_{13}CH_3$, $SiCH_2CH_2CH_2NH$), 2.51 (t, J=6.6 Hz, 2H, $NCOCH_2$), 2.52 (t, J=6.6 Hz, 2H, $NCOCH_2$), 3.19~3.29 (m, 6H, $NCH_2CH_2(CH_2)_{13}CH_3$, $SiCH_2CH_2CH_2NH$), 3.80 (q, J=7.3 Hz, 6H, $SiOCH_2CH_3$), 6.40 ppm (br,1H, NHCO).
